# Supplementary material for: “A man never cries”: A mixed-methods analysis of gender differences in depression and alcohol use in Moshi, Tanzania
Source: PLOS Glob Public Health. 2025 Feb 28;5(2):e0002664. doi: 10.1371/journal.pgph.0002664 (PMC11870386; doi:10.1371/journal.pgph.0002664)
Supplement: Attachment — Submitted filename: A Man Never Cries.docx [file pgph.0002664.s003.docx]

A Man Never Cries”: A Mixed-Methods Analysis of Gender Differences in Depression and Alcohol Use in Moshi, Tanzania

| Line | Present | Comment |
| --- | --- | --- |
| 75 | No key word | To add key word |
| 136 | Method | To be summarized in proper way |
| 300 | Relation ship | To use correlation |
| 301 | Linear relation | Is it positive relation or negative relation |
| 310 | Linear relation figure | Not available |
| 469 | Our | Better to avoid use our, we |
| 489 | Our results | Not use our |
| 578 | Reference | What is style |
| 581 | Reference | What is style |
| 587 | Reference | What is style |
