# Supplementary material for: “A man never cries”: A mixed-methods analysis of gender differences in depression and alcohol use in Moshi, Tanzania
Source: PLOS Glob Public Health. 2025 Feb 28;5(2):e0002664. doi: 10.1371/journal.pgph.0002664 (PMC11870386; doi:10.1371/journal.pgph.0002664)
Supplement: Attachment — Submitted filename: Response to Reviewers - Mental Health.docx [file pgph.0002664.s005.docx]

March 28th, 2024

To Whom It May Concern:

We appreciate the constructive suggestions presented to us by our reviewers and thank them for the opportunity to improve our manuscript. We have incorporated the changes requested by both reviewers in our attached submission with our specific modifications outlined below. Our revised manuscript is attached to this resubmission packet.

Reviewer #1:

**Comment**: Follow a reporting guideline such as STROBE so that no comments are missing in reporting.

*Response: We thank the reviewer for this feedback. We have reviewed the STROBE guidelines and have made the following changes. 1. We added in sample size calculation information for our quantitative data collection methodologies. 2. We added a sentence to the quantitative data analysis to state that missing data for our measured variables were extremely limited. Missing values are noted in Table 1 and Table 2. 3*. *Finally, we added a sentence in the quantitative results section to highlight the total number of participants enrolled and then analyzed describing the difference between these two values. All other STOBE guidelines have been previously addressed in the paper.*

**Comment**: Please report the reliability and validity of the tool that the author used.

*Response: We thank the reviewer for this feedback. The DrInC, AUDIT, and PHQ-9 tools used in this study have been validated (culturally and psychometrically) in Tanzania. The validated versions of these tools were used in this study and noted in the instruments section of our quantitative data collection methods. The questionnaire was developed and validated through pilot testing and editing as necessary with the study team. More information on the questionnaire development is reported in the response below.*

**Comment**: The questionnaire development and validation are required to be reported.

*Response: We thank the reviewer for this feedback. We have added the following information on the development of the quantitative questionnaire to our manuscript under the Quantitative Data - Instruments portion of our methods section: “Quantitative surveys consisted of five main components: (1) basic demographic data, (2) self-reported alcohol use data consisting of scaled multiple choice or binary yes-no questions, (3) the Alcohol Use Disorder Identification Test (AUDIT), (4) the Drinkers’ Inventory of Consequences (DrInC), and (5) the nine-item Patient Health Questionnaire (PHQ-9).”*

*Further, we have provided a greater explanation of the validation of the questionnaire. This addition reads, “The first and second components were developed through an extensive literature review and research team expertise developed through years of alcohol-based research in the region. The remaining components including the AUDIT, DrInC, and the PHQ-9 are reliable and validated measures. The tool was pilot-tested in the local language of Kiswahili and appropriate changes were made to ensure the validation and cultural relevance of the survey instrument.”*

**Comment**: Stressing PHQ-9 and AUDIT throughout the manuscript creates ambiguity, so it is recommended to use depression.

*Response: We thank the reviewer for this feedback. PHQ-9 and AUDIT scores have been used consistently within the results section to provide a more precise and accurate representation of patients’ alcohol use, and anxiety- and depression-related symptoms, given that both measures are reported on an increasing rather than a dichotomous scale. To maintain this accuracy within our results section, we have opted to maintain our usage of PHQ-9 and AUDIT. Within the discussion section, we use the terminology, ‘Major Depressive Disorder (MDD)’ instead of reporting PHQ-9 scores.*

**Comment**: The statistical model requires an explanation of why it was used.

*Response:*

*We thank the reviewer for bringing this gap in our manuscript to our attention. A linear regression model was used to assess the relationship between two quantitative variables: PHQ-9 as the dependent variable and AUDIT as the independent variable. Our rationale for using this statistical model is grounded in existing literature, which highlights the frequent co-occurrence of depression and AUD. Specifically, higher PHQ-9 scores often indicate more severe depression, while elevated AUDIT scores signify harmful drinking behaviors (e.g., AUD). However, in the context of Moshi, Tanzania, this linear relationship between PHQ-9 and AUDIT remains largely unexplored. Therefore, we opted for this statistical approach to bridge this research gap and evaluate the strength and direction of this relationship within this study population.*

*Additionally, the regression coefficients provided insight into the average change in PHQ-9 scores associated with a one-unit change in AUDIT scores. This is clinically significant when assessing the risk of depression among individuals with AUD. As such, we have updated the ‘Quantitative Data - Analysis’ portion of our methods section to read, “Due to the frequent co-occurrence of depression and AUD in other LMIC regions, this statistical model was selected to examine the linear relationship between PHQ-9 and AUDIT within our study population, specific to Moshi, Tanzania.”*

*Finally, upon re-evaluation of our methods, we identified an error in our initial description of the statistical method used to assess the relative risks across different predictors of depression (e.g., Female Gender, ED Women, RHC Women, ED Men, HHD Status, and Sought Psychiatric Treatment). Contrary to what was stated in our manuscript, we did not employ a log-binomial regression. Instead, we utilized contingency tables to calculate unadjusted relative risks. This approach was used because it offers a straightforward and interpretable way to compare unadjusted risk ratios of the outcome variable (e.g., MDD Status) across different categories of independent, binary predictor variables among our study population. We have revised our manuscript accordingly.*

**Comment**: All the assumptions must be met before fitting the model. And they must be reported.

*Response:*

*We thank the reviewer for this suggestion and the opportunity to improve our manuscript in addressing it. Among all three models (e.g., ED men, RHC women, and ED women), the assumptions of linearity, independence, homoscedasticity, and normality of residuals were only met for the model assessing the linear relationship of AUDIT and PHQ-9 scores among ED men. We have updated the ‘Quantitative Data - Analysis’ portion of our methods section as such. This addition reads, ‘All assumptions were met for the model assessing the linear relationship between PHQ-9 and AUDIT scores among ED Men, meaning that (1) there were no obvious departures from linearity as observed via scatter plot examination of the independent versus dependent variable, (2) independence of observations was assumed, given that observations were independent of each other, (3) homoscedasticity was assessed by plotting the residuals against the fitted values, with no discernable pattern, and (4) normality was evaluated and confirmed using inspection of the Normal Q-Q Plot, with residuals following the diagonal line. These assumptions were met only for the linear regression model.’*

*As stated previously, we acknowledge the error in our initial description of the statistical method used to quantify the relative risk of experiencing MDD across different predictors. Contrary to what was stated in our manuscript, we did not employ a univariate log-binomial regression. Instead, we utilized contingency tables to calculate unadjusted relative risks, which was an acceptable approach given the binary nature of our outcome (e.g., MDD) and independent predictor variables (e.g., Female Gender, ED Women, RHC Women, ED Men, HHD Status, and Sought Psychiatric Treatment). Observations were independent of each other, and the sample size was adequate given the examination of cell frequencies. To assess potential confounding variables, we examined their distribution across levels of the different predictor variables.*

**Comment**: If separate models such as linear regression and logistic regression were used, their assumptions would not be the same. The RR as an estimate needs to be checked, as the estimate from the binary regression is the odds ratio.

*Response: We thank the reviewer for this feedback. To clarify, we used contingency tables to calculate unadjusted relative risks of MDD across different independent predictor variables (e.g., Female Gender, ED Women, RHC Women, ED Men, HHD Status, and Sought Psychiatric Treatment), which provides unadjusted estimates of relative risk (RRs), quantifying the relationships between the independent, binary predictor variables and MDD status. No adjusted relative risks were calculated. These corrections have been made in the manuscript which read, “Contingency tables, in combination with the ‘epiR’ R package, were used to calculate unadjusted relative risk (RR) of the binary outcome variable (e.g., MDD status) across different, independent, binary predictors of MDD.”*

**Comment**: All the models' fitness should be reported. The model's fitness can be plotted. The plots are different for each model. Please report the plots.

*Response: Thank you for this feedback. Please refer to Figure 1: Linear Regression between PHQ-9 and AUDIT Scores across Patient Populations, where the adjusted R-squared value is provided for each model, indicative of each model’s fitness. Additionally, please refer to the last paragraph of the Quantitative Results section, where we report the results of each linear regression model, along with their respective adjusted R-squared values.*

Reviewer #2:

**Comment**: Line 75 No key word To add key word

*Response: We thank the reviewer for bringing this to our attention. Key words have been added in line 76 below the abstract. These include “alcohol use, depression, mental health, gender, Tanzania”.*

**Comment**: Line 136 Method To be summarized in proper way

*Response: We appreciate this feedback. In line with feedback from Reviewer #1, we edited the document to ensure all STROBE reporting guidelines were met and summarized appropriately. All relevant and required information is in the methods section.*

**Comment**: Line 300 Relation ship To use correlation

*Response: We appreciate this feedback. In this sentence, we have replaced the term ‘relationship’ with ‘correlation’.*

**Comment**: Line 301 Linear relation Is it positive relation or negative relation

*Response: We thank the reviewer for bringing this gap to our attention. We have updated this sentence to read, “...AUDIT and PHQ-9 scores were found to be positively and linearly associated only among ED men.”*

**Comment**: Line 310 Linear relation figure Not available

*Response: We thank the reviewer for this feedback. We have attached Figure 1, displaying the linear regression analysis between PHQ-9 and AUDIT scores, to this resubmission packet.*

**Comment**: Line 469 Our Better to avoid use our, we

*Response: Thank you, we have updated the language to “Key findings from this analysis were…”.*

**Comment**: Line 489 Our results Not use our

*Response: Thank you, we have updated the language to “The results..”.*

**Comment**: Line 578 Reference What is style

*Response: We appreciate this comment from the reviewer. This is the Vancouver reference style, the same citation format used for the rest of our references. The reason for the slightly different style appearance is that in this article, the authors are listed as one entity, “GBD global collaborators.”*

**Comment**: Line 581 Reference What is style

*Response: We appreciate this question. As detailed above, this is the Vancouver reference style.*

**Comment**: Line 587 Reference What is style

*Response: We appreciate this question. As detailed above, this is the Vancouver reference style.*
