# Supplementary material for: “A man never cries”: A mixed-methods analysis of gender differences in depression and alcohol use in Moshi, Tanzania
Source: PLOS Glob Public Health. 2025 Feb 28;5(2):e0002664. doi: 10.1371/journal.pgph.0002664 (PMC11870386; doi:10.1371/journal.pgph.0002664)
Supplement: Attachment — Submitted filename: Response to Reviewers - Mental Health revision 2.docx [file pgph.0002664.s006.docx]

December 18th, 2024

To whom it may concern,

We very much appreciate the helpful suggestions and methodological questions raised by our reviewers and thank them for the opportunity to improve our manuscript further. We have incorporated the requested changes. Our specific modifications are outlined below, and our revised manuscript has been attached to this resubmission packet.

**Comment:** Reviewer #2: thanks for your efforts

*Response: We also thank you and appreciate all your helpful suggestions.*

**Comment:** Reviewer #3: Line 140: The type of study design for the survey is to be stated. For cross-sectional study design, RR is not suitable to be used unless if the study outcome is rare (OR can be interpreted similarly to RR). In this case unlikely.

*Response: We thank the reviewer for this helpful comment. We have changed the ‘Study Design Overview’ section of our manuscript to provide greater detail on our study design. This now reads: “Between October 2021 and May 2022, data were collected to explore gender differences in alcohol consumption in Moshi, Tanzania, an urban town of over 200,000 residents. This study employed a cross-sectional, mixed-methods, sequential explanatory approach, with all data collected at the Kilimanjaro Christian Medical Center (KCMC), a large referral hospital in the region that provides care to over 1.9 million people [41].*

**Comment:** Line 151/253: What if the sample size of 20 is not sufficient for research study to reach saturation?

*Response: We thank the reviewer for this question and have made modifications to both the ‘Study Design Overview’ and the ‘Qualitative Data - Sampling’ sections to address this. Our final sample size was guided by both qualitative research conventions and thematic saturation, and was flexible enough to ensure that the latter was met.*

*In our ‘Study Design Overview’ section, we have revised our previous statement to now read “Out of these enrolled patients, a purposive sampling approach was used to select individuals for participation in IDIs until saturation was reached. The qualitative findings from the final 19 participating individuals allowed for a deeper exploration of how gender impacts the overall relationship between mental health and risky alcohol use behaviors.”*

*We have also fully re-written our ‘Qualitative Data - Sampling’ section to better explain our approach. Our revised paragraph reads: “Of the 655 individuals with fully completed surveys, the study team aimed to recruit up to 20 participants for participation in IDIs or until thematic saturation was reached, whichever came first. This dynamic approach allowed us to ensure balanced representation while also leaving thematic saturation as the ultimate determinant for data collection. Our initial target sample size was based on widely accepted qualitative research guidelines recommending 15-20 interviews for saturation with narrow study aims and a relatively homogenous group [54]. Thematic saturation was defined as the lack of new themes or information after three consecutive interviews. To obtain a gender-balanced perspective, the team aimed to interview five female ED patients, five female RHC patients, and ten male ED patients, ceasing once saturation was reached, which occured for ED males after nine interviews, leaving 19 IDIs collected in total.”*

**Comment:** Line 153: Inclusion criteria is to be stated.

*Response: We thak the reviewer for this suggestion. We have changed this section to be entitled, ‘Eligibility Criteria’ and have revised it accordingly. This change now reads: “Participants were eligible for study participation if they were aged 18 years or older, fluent in KiSwahili, and were receiving care at KCMC’s RHC or ED. Participants were excluded from the study if they were unable to provide informed consent, or were not clinically sober, medically stabilized, or well enough to complete the survey verbally on their own.” We hope that these changes address the reviewer’s concern.*

**Comment:** Line 206: Analysis is to be replaced with Statistical Analyses.

*Response: We appreciate this suggestion and have changed the manuscript accordingly.*

**Comment:** Line 213: Studying the relationship between AUDIT and PHP-9 alone is not sufficient. The outcome can be contributed by other/confounding factors.

*Response: We appreciate this comment from the reviewer. In addition to examining the unadjusted association between PHQ-9 scores and AUDIT scores, we conducted adjusted analyses to control for potential confounding factors. Specifically, we fit an adjusted log-binomial regression model that included age, employment, marital status, female gender, and clinical group as covariates, identified as significant predictors in our preliminary unadjusted analyses. We have revised our manuscript accordingly, which now states: "Potential confounding factors, including age, employment, marital status, gender, and clinical group, were also assessed in both models. Significant unadjusted predictors identified through preliminary analyses were further included in adjusted models to control for these confounders."*

**Comment:** Line 215: Can the relationship be the other way round e.g. AUDIT as dependent and PHQ-9 as predictor or a bilateral relationship?

*Response: We thank the reviewer for bringing this to our attention. In response to the reviewer’s suggestion, we tested the robustness and potential bidirectionality of the relationship. We conducted an additional linear regression analysis treating AUDIT as the dependent variable and PHQ-9 scores as an independent variable. This reverse modeling provided further evidence that the relationship between depression and hazardous drinking is not merely unidirectional, which we have noted in the revised manuscript. The revised manuscript now reads, "To evaluate this further, we conducted a reverse regression model, treating AUDIT scores as the dependent variable and PHQ-9 scores as the independent variable. This allowed us to examine the robustness and bidirectionality of the relationship between depression and hazardous drinking."*

**Comment:** Line 230: What about other variables like age, employment, marital status?

*Response: We appreciate this question from the reviewer and have better described what variables have been tested, with explicit mention of age, employment status, marital status, female gender, and clinical group as potential confounders, tested in both unadjusted and adjusted models.*

**Comment:** If the variables in the unadjusted analyses were found to be significant, adjusted analysis could be done.

*Response: We thank the reviewer for this comment and agree. We would like to better clarify our quantitative analysis approach. First, we initially fit log-binomial regression models to identify significant univariate predictors of MDD Status (PHQ-9 ≥ 10). All variables that showed statistically significant associations in unadjusted analyses were subsequently included in adjusted models, including age, employment, marital status, female gender, and clinical group. This stepwise approach ensured that we only included relevant confounders in the final adjusted models, thereby improving the validity and interpretability of our findings. Our revised manuscript now reads, "Significant unadjusted predictors identified through preliminary analyses were further included in adjusted models to control for these confounders."*

**Comment:** Line 231: The version of the software is to be mentioned.

*Response: We thank the reviewer for bringing this gap in our methods to our attention, and have added the software version: RStudio (version 1.4.1106).*

**Comment:** Line 232: Which variable that was adjusted using Mantel-Haenszel is to be mentioned.

*Response: We thank the reviewer for this comment. Upon further reflection, we realized that the Mantel-Haenszel approach was initially considered, but ultimately, we employed a log-binomial regression model to produce adjusted RRs. The covariates of age, employment status, marital status, gender, and clinical group were tested as potential confounders and adjusted for this analysis. Therefore, we have removed references to Mantel-Haenszel adjustments and clarified that we used a generalized linear model (log link) to control for confounders. The revised manuscript states: "For adjusted RRs, we employed a generalized linear model with a log link (log-binomial regression) to control for confounding variables. Significant unadjusted predictors such as age, employment status, marital status, female gender, and clinical group, identified through preliminary analyses, were included as covariates in the adjusted model."*

**Comment:** Line 239: The acceptance significance level of p value, one or two-tailed test p value is to be stated.

*Response: We appreciate the reviewer in bringing this gap to our attention. We have updated our methods section accordingly to note the use of a two-tailed test.This revision reads: "the acceptance significance level for all statistical tests was set at p<0.05, and two-tailed tests were applied."*

**Comment:** For qualitative method, please state if all the interviews were conducted face to face. Were the interviews recorded?

*Response: We appreciate this suggestion and have revised the ‘Procedures’ section of our qualitative methods to address these gaps. This addition reads: “All IDIs were conducted face-to-face with the participant and the same gender-matched research assistant who had enrolled then previously. All interviews were audio-recorded and conducted in a private, unoccupied room at KCMC.”*

**Comment:** Line 292-293: The reference number is to be provided.

*Response: We have added the reference numbers for all ethical approval certificates as requested. The sentence previously on like 292-293 now reads: “Ethical approval for this study was obtained from the Duke University Institutional Review Board (Pro00107861), the Tanzanian National Institute of Medical Research (NIMR/HQ/R.8a/Vol. IX/3734), and the Kilimanjaro Christian Medical University College Ethical Review Board (No. 2515) prior to the onset of any data collection.”*

**Comment:** Table 1, 2, 3: The statistical tests employed here is to be denoted in table footnote. If there are additional statistical tests used for these tables, it is to be mentioned in the statistical analysis section (Line 206). For Mantel-Haenszel test, indicate which variable was adjusted.

*Response: We thank the reviewer for bringing this gap in our methods and results to our attention. We have added footnotes to tables 1, 2, and 3 to clarify which statistical tests were used, and have added the following description in our ‘Statistical Analysis’ section: “Continuous demographic, PHQ-9, and AUDIT variables underwent ANOVA testing to compare means across our multiple patient groups, while categorical variables were assessed through Chi-squared test.” Contingency tables, in combination with the “epiR” R package (version 2.0.19) [53], were used to first calculate unadjusted relative risks (RR) for binary outcomes (e.g., MDD status) across key independent binary predictors, which we note in the manuscript.*

*As stated previously, references to Mantel-Haenszel have been removed since this method was not ultimately used; instead, we employed a log-binomial regression model to produce adjusted RRs, adjusting for female gender, clinical group, age, employment, and marital status. The manuscript now reads, “For adjusted RRs, we employed a generalized linear model with a log link (log-binomial regression) to control for confounding variables. Significant unadjusted predictors such as age, employment status, marital status, female gender, and clinical group, identified through preliminary analyses, were included as covariates in the adjusted model”*

**Comment:** For the results section, the decimal point for percentage figures in the text is to be at least 1 decimal point and consistent.

*Response: We thank the reviewer for this comment. We have gone through our results section to provide at least 1 decimal point for percentages and have ensured this is consistent across our manuscript.*

**Comment:** P Value to be replaced with p= or p<

*Response: We appreciate this comment and have made these changes.*

**Comment:** Line 325-329: The word correlation refers to r (correlation coefficient). R² (coefficient of determination) does not directly represent the correlation but rather how much of the variance in PHQ-9 scores is explained by AUDIT scores. If correlation is reported, r instead of R² is to be used.

*Response: We thank the reader for helping us to better clarify our results. We have made changes to our manuscript accordingly. This change now reads: “Finally, in examining the relationship between alcohol consumption and depressive symptoms, a positive linear association was observed between AUDIT and PHQ-9 scores only among ED men (r = 0.33, R² = 0.11; p<0.001) (Figure 1). This indicates that approximately 11% of the variance in PHQ-9 scores is explained by AUDIT scores in this group. No significant linear association was found between depression and alcohol use among ED women (r = 0.04, R² = 0.002; p=0.668) or RHC women (r = 0.14, R² = 0.02; p=0.014).”*

**Comment:** For Table 3: the frequency for predictors to be presented before deriving the RR.

*Response: We thank the reviewer for bringing this to our attention. We have revised Table 3 to include the frequency of MDD Status (e.g., MDD prevalence) among the different predictors prior to reporting the relative risks (RR).*

**Comment:** Missing data information is to be provided/stated if any.

*Response: We thank the reviewer for this feedback and have provided information on missing data in our quantitative results section. This addition reads: “Data was missing only from the variable ‘sought psychiatric treatment’ analyzed in Tables 2 and 3, and these were excluded from the analysis.” We hope this addresses the readers concern. Information on missing data is also mentioned in the ‘statistical analysis’ section of our results.*

**Comment:** For the qualitative analysis, the study ID of the participants could be used to indicate their responses.

*Response: We thank the reviewer for this suggestion. We have provided the IDI number for all quoted participants in our qualitative results.*

**Comment:** Revisit the STROBE checklist and ensure all the information stated there are highlighted in the manuscript.

*Response: We thank the reviewer for this comment. We have added a dedicated section on sample size estimations based on the STROBE checklist. This addition reads: “The sample size for the quantitative analysis was determined based on the expected difference in the Alcohol Use Disorders Identification Test (AUDIT) scores between the RHC and ED populations. Using insights from existing studies and input from local research team members, we estimated the prevalence of AUD to be 10% in women at the RHC, 15% in women at the ED, and 30% in men at the ED. With 80% power and 90% confidence, we calculated that a sample size of 587 participants would be sufficient to assess the prevalence of risky alcohol consumption across the three study groups. Since the anticipated difference in AUD prevalence was smaller between the two female populations than between males and females, the research team initially prioritized enrolling more women to ensure adequate statistical power for detecting differences between the female groups.*

*When preliminary analysis revealed that the AUD prevalence was considerably higher than expected (40% in ED women and 45% in ED men), we recalculated the required sample size. Based on these revised prevalence estimates, 1,200 participants would be needed to detect a meaningful difference in proportions between these groups. However, given constraints in study time and funding, it was not feasible to enroll this larger sample. With IRB approval, the study target was adjusted to recruit as many male and female participants as possible within the available timeline.*

*Additionally, we have ensured all other items from the STROBE checklist are present in our manuscript.*
